# Mitochondrial DNA efflux as a potential amplifier of systemic inflammatory network rewiring in heart failure with preserved ejection fraction

**DOI:** 10.3389/fimmu.2026.1866184

**Published:** 2026-06-22

**Authors:** Xingwei Zhao, Shengyu Huang, Qiulin Li, Yang Yu, Chunxiang Zhang

**Affiliations:** 1 Key Laboratory of Medical Electrophysiology, Ministry of Education and Medical Electrophysiological Key Laboratory of Sichuan Province, Institute of Cardiovascular Research, Southwest Medical University, Luzhou, Sichuan, China; 2 Department of Anesthesiology, The Affiliated Hospital, Southwest Medical University, Luzhou, Sichuan, China; 3 Department of Cardiology, The Affiliated Hospital of Southwest Medical University, Luzhou, Sichuan, China

**Keywords:** cGAS-STING pathway, cross-organ communication, extracellular vesicles, heart failure with preserved ejection fraction (HFpEF), innate immune recognition, mitochondrial DNA (mtDNA), network remodeling, systemic inflammation

## Abstract

Heart failure with preserved ejection fraction (HFpEF) is a systemic inflammatory disease that affects multiple organs. However, the integration of different comorbid stress factors into a persistent and organ-specific inflammatory network remains unclear. Under the background of HFpEF, mitochondrial DNA (mtDNA) may not only play a role as a damage-associated molecular pattern (DAMP), but also act as a cross-organ inflammatory signal, linking the comorbid-driven mitochondrial stress with endothelial dysfunction, myocardial remodeling, and extracardiac organ involvement. Under the influence of HFpEF-related stress factors, including aging, obesity, diabetes, hypertension, and renal dysfunction, mtDNA may undergo oxidation and structural remodeling and be released in the form of free DNA, extracellular vesicle (EV)-related DNA, or neutrophil extracellular trap-related DNA. These mtDNA signals may activate the nucleic acid sensing pathways mediated by TLR9 and cGAS-STING, and promote the activation of downstream NLRP3 inflammasomes in endothelial cells, cardiomyocytes, fibroblasts, immune cells, and extracardiac tissues, thereby promoting IL-6/TNF production, type I interferon signaling, inflammasome activation, and self-amplifying inflammatory circuits related to the progression of HFpEF. Within this framework, HFpEF can be understood as a cross-organ network reconfiguration state, where mtDNA-related inflammatory signals may lead to abnormal information flow, especially in the internal phenotype characterized by metabolic stress, age-related mitochondrial damage, renal dysfunction, and systemic inflammation. The coupling between mtDNA generation, transmission, and decoding may amplify endothelial dysfunction, myocardial stiffness, fibrosis, and phenotypic-specific inflammatory remodeling; however, these processes occur within a broader pathological biology background of HFpEF, which also includes mechanisms independent of mtDNA, such as impaired NO–cGMP–PKG signaling, low phosphorylation of myosin, vascular stiffness, renal dysfunction, neurohumoral activation, and extracellular matrix remodeling. Therefore, this article proposes that mtDNA efflux is an inflammation amplifier that depends on the phenotype and disease stage, rather than being a universal or unique mechanism for explaining all HFpEF phenotypes. The existing evidence does not yet prove that mtDNA efflux is the main causal driver of HFpEF; instead, its position in the temporal sequence and causal relationship still needs to be verified in longitudinal studies and intervention studies specific to HFpEF.

## 1 Introduction

HFpEF has gradually been recognized as a systemic disease state shaped by metabolic disorders, vascular aging, immune activation, and multi-organ imbalance, rather than merely a disorder of diastolic function. HFpEF is continuously accompanied by a low-grade, chronic, and organ-wide inflammatory response that affects multiple systems such as blood vessels, adipose tissue, skeletal muscle, and kidneys (1). However, a key question remains unanswered: How can the combined stress from different sources be integrated into a sustainable, transmissible, and organ-specific inflammatory network? Existing models, whether emphasizing endothelial dysfunction, metabolic inflammation, or cumulative comorbidity burden, can explain local pathological changes but struggle to explain how inflammation spreads between organs, why it can persist for a long time, and how it eventually stabilizes as diverse HFpEF phenotypes. From this perspective, HFpEF should be understood as a systemic inflammatory network imbalance rather than a single organ or pathway abnormality. Therefore, understanding HFpEF requires not only a clear understanding of the established local mechanisms, such as endothelial dysfunction, metabolic inflammation, renal function impairment, vascular stiffness, and extracellular matrix remodeling, but also the identification of the signaling molecules that may connect these mechanisms between cells and organs. Accordingly, the following discussion takes HFpEF as the main organizational framework, and only discusses mtDNA biology within the scope that is helpful for explaining the systemic inflammation, microvascular dysfunction, myocardial remodeling, and interorgan perturbations related to HFpEF.

Mitochondrial DNA (mtDNA) provides an important entry point to this issue. As a nucleic acid molecule that retains the ancestral characteristics of bacteria, mtDNA is rich in unmethylated CpG sequences and undergoes structural and chemical modifications under oxidative stress conditions. This enables the ectopically located mtDNA to activate nucleic acid sensing pathways such as TLR9 and cGAS-STING, and at the same time, promotes the activation of downstream NLRP3 inflammasome under conditions such as mitochondrial stress, generation of reactive oxygen species (ROS), ion imbalance, or accumulation of oxidized mtDNA (2). More importantly, under metabolic, mechanical, and inflammatory stress conditions, mtDNA is not confined within the mitochondria but can be released into the cytoplasm and further enter circulation, existing in various forms such as free DNA, extracellular vesicles(EVs), neutrophil extracellular traps(NETs), or protein complexes (3–5). These characteristics indicate that under pathological conditions, mtDNA may be transmitted between cells and organs, thereby serving as a potential inflammatory input that transmits local mitochondrial damage signals to distant tissues.

In HFpEF, mtDNA may function as a damage-associated molecular pattern and a systemic inflammatory signal, linking multiple comorbidity-related stress factors with persistent inflammation in the endothelium, myocardium, immune system, kidneys, adipose tissue, and skeletal muscle (2, 8). Under stress conditions, mtDNA can be damaged, oxidized, fragmented, or released from the mitochondria, thereby forming different molecular states with varying immune activities (27). These mtDNA signals can then be transmitted at the cellular and systemic levels as free mtDNA, extracellular vesicles (EV)-associated mtDNA, or neutrophil extracellular traps (NET)-associated mtDNA (21, 25). Finally, mtDNA can be recognized by the hierarchical innate immune system, mainly through the TLR9 in the endolysosomal compartment and the cGAS-STING pathway in the cytoplasm; meanwhile, NLRP3, as the downstream inflammasome platform, amplifies the inflammatory response under conditions such as mitochondrial dysfunction, ROS generation, changes in ion flux, and accumulation of oxidative or ectopic mtDNA (27, 35). Meanwhile, these inflammatory responses may further aggravate mitochondrial damage and promote more mtDNA release, thereby possibly forming a self-reinforcing inflammatory feedback loop (9, 10).

In the context of this chronic, low-grade inflammatory state of HFpEF, this information flow process holds special significance. Unlike the brief signal bursts in acute inflammation, HFpEF relies more on low-level but continuous mtDNA input and its circulation transmission between multiple organs (6, 7).This characteristic enables it to integrate various comorbidity stresses such as obesity, diabetes, aging, and renal dysfunction and stabilize as a clinically heterogeneous phenotype. Therefore, within this framework, the inflammatory pathophysiology associated with HFpEF should not be regarded as the result of a single abnormal inflammatory pathway, but rather as a systemic and network-level process. During this process, the released and circulating mtDNA can, depending on its source, oxidation state, transport form, and cellular environment, activate the TLR9 and cGAS-STING nucleic acid sensing pathways differently, and promote the downstream NLRP3 inflammatory body amplification effect across organs, thereby participating in endothelial, myocardial, immune and metabolic remodeling. Based on the above understanding, this article will start from the continuous process of “generation and encoding - cross-zone compartmental transmission - hierarchical perception and signal amplification - organ-specific response - cross-organ network integration”, systematically explore the mechanism of mtDNA in HFpEF, and further discuss its potential significance in disease classification and treatment strategies. Therefore, this review is not intended to provide a general overview of mtDNA immunology; rather, its focus is on exploring how mtDNA-related inflammatory signals may potentially contribute to the maintenance, amplification, heterogeneity, and potential therapeutic targeting of HFpEF. Importantly, this review does not consider mtDNA efflux as a single unified mechanism to explain the entire HFpEF spectrum. The heterogeneity of HFpEF stems from multiple overlapping but not entirely identical mechanisms, including endothelial and coronary microvascular inflammation, impaired NO–cGMP–PKG signaling, low myosin phosphorylation, extracellular matrix expansion, decreased metabolic flexibility, dysfunction of adipose tissue, renal function impairment, neurohumoral activation, vascular stiffness, and age-related immune dysregulation.

**Figure 1:**
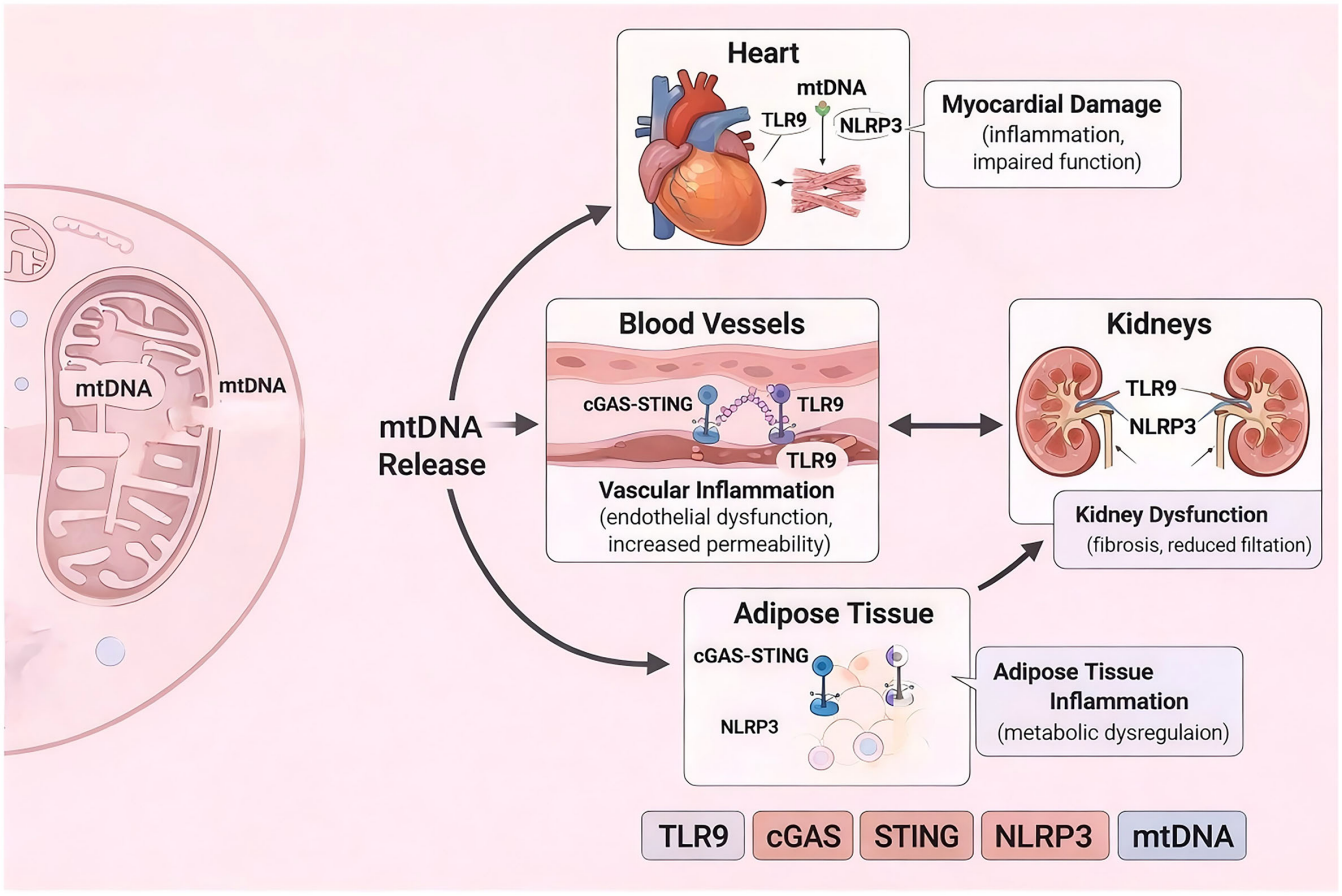
Framework of the mtDNA-associated systemic inflammatory network in HFpEF. This figure illustrates the overall framework of an mtDNA-associated cross-organ inflammatory network in HFpEF. On the left is the mitochondrial structure, and mtDNA is released into the cytoplasm and extracellular environment under conditions of cellular stress or damage (“mtDNA release”). The released mtDNA spreads through the circulatory system (central arrow) and acts on multiple target organs. In the vascular system (middle part), mtDNA is recognized by the cGAS–STING pathway and TLR9 in endothelial cells, inducing vascular inflammatory responses, manifested as endothelial dysfunction and increased permeability (vascular inflammation). In the heart (upper part), mtDNA is perceived through the TLR9 and NLRP3 inflammatory pathway, leading to myocardial inflammation and functional impairment (myocardial damage). In the kidneys (right), mtDNA also activates the TLR9 and NLRP3 signaling pathways, promoting fibrosis and decreased filtration function (kidney dysfunction). In adipose tissue (below), mtDNA induces inflammatory responses through the cGAS–STING and NLRP3 pathways, causing metabolic disorders (adipose tissue inflammation). The solid arrows in the figure represent the direction of mtDNA release from mitochondria and its spread to different organs, while the double arrows indicate the interaction and inflammatory signal communication between organs. The activation of the nucleic acid sensing pathways mediated by TLR9 and cGAS-STING, as well as the amplification effect of the NLRP3 inflammasome, may jointly promote the formation of a cross-organ positive feedback inflammatory network and participate in the progression of HFpEF, especially in inflammatory, metabolic, senescence-related, and cardiorenal types. The bottom legend indicates the color coding of the main molecular pathways: mtDNA, mitochondrial DNA; HFpEF, heart failure with preserved ejection fraction; TLR9, Toll-like receptor 9; cGAS, cyclic GMP-AMP synthase; STING, stimulator of interferon genes; NLRP3, NOD-like receptor family pyrin domain containing 3.

In this context, mtDNA outflow should best be regarded as a supplementary framework that can link mitochondrial damage with innate immune activation within specific HFpEF phenotypes, rather than replacing established non-mtDNA mechanisms. Given the current limitations in causal evidence, the terms used in this review are intentionally cautious. We do not assume that mtDNA outflow is the primary initiating driving factor of HFpEF. Instead, we position mtDNA-related signals as a candidate mediator factor and a phenotypically-dependent inflammation amplifier, whose role may vary depending on the disease stage, comorbidity background, tissue compartment, and HFpEF phenotype. Whether mtDNA outflow occurs before the onset of HFpEF or mainly reinforces the already formed inflammatory remodeling remains an important question that requires future longitudinal studies and intervention research to answer. The framework of the mtDNA-associated systemic inflammatory network in HFpEF is shown in **Figure 1**.

## 2 mtDNA release and propagation in HFpEF-related systemic inflammation

### 2.1 mtDNA immunogenicity in HFpEF-related sterile inflammation

In HFpEF, the relevance of mtDNA immunogenicity lies in the possibility that it may have the ability to transform chronic comorbid-related mitochondrial stress into persistent sterile inflammation. The reason why mitochondrial DNA (mtDNA) can trigger an inflammatory response lies in its dual characteristics of “non-selfness” and “plasticity signal attributes” in terms of evolutionary origin and structural features. As a nucleic acid molecule derived from the ancestors of bacteria, mtDNA is rich in unmethylated CpG sequences and lacks the histone-mediated shielding effect. When mtDNA undergoes ectopic localization, it can be recognized by nucleic acid sensors such as TLR9 in the endolysosomal compartment and cGAS in the cytoplasm, thereby activating the cGAS-STING pathway. In contrast, NLRP3 should not be regarded as a direct mtDNA receptor; instead, it functions as a platform for the formation of inflammasomes and a downstream amplifier of inflammation, and can be activated in the context of mitochondrial stress, reactive oxygen species (ROS) generation, changes in ion flux, lysosomal damage, and the presence of oxidative or ectopic mtDNA (8). However, an increasing number of studies suggest that the immune activity of mtDNA is not a static property but more akin to a “signal encoding process” that can be regulated (9, 10).

During this encoding process, the oxidative modification and structural remodeling of mtDNA play a potential role. Under oxidative stress conditions, mtDNA can undergo 8-oxo-dG modification, fragmentation, and topological conformational changes, which significantly affect its immune recognition properties. For instance, oxidative modification can enhance the binding affinity of mtDNA to cGAS, thereby amplifying the activation of the downstream TBK1-IRF3 signaling axis (11).Xian et al (12). demonstrated, by inducing mPTP opening and VDAC oligomerization in primary macrophages and combining with the OGG1/FEN1 deficiency model, that oxidized mtDNA is processed into a fragment of approximately 500–650 bp and released into the cytoplasm; further studies revealed that these oxidized fragments can induce a more persistent type I interferon response and lower the activation threshold of NLRP3 inflammasome (12).These results collectively indicate that mtDNA can function as a processed inflammatory input, specifically by releasing oxidized-modified fragments (500–650 bp) into the cytoplasm through BAX/BAK and VDAC-dependent channels. Its immunological activity depends on the degree of oxidation, fragment length, and structural conformation.

Therefore, from the perspective of information theory, the immunogenicity of mtDNA can be understood based on its oxidation state (such as 8-oxo-dG), fragment length, and spatial/topological conformation; these factors jointly determine the “strength and nature of the encoded signal”, rather than merely existing or not existing in a simple binary form.

In addition to the “encoding” at the molecular level, the immunogenicity of mtDNA is also strictly regulated by spatial compartments. Under normal circumstances, mtDNA is restricted within the mitochondria and in an immune-isolated state; once it enters the cytoplasm or the extracellular environment, it rapidly transforms into a recognizable danger signal (9). Caielli et al.’s research showed that monocytes, after phagocytosing red blood cells containing mitochondria, can induce the release of endogenous mtDNA into the cytoplasm through the RLR-MAVS pathway and trigger an inflammatory response without relying on cell pyroptosis (13). This discovery indicates that the release of mtDNA is not a passive leakage, but rather an active, regulated and selective release process. This process is mediated by BAX/BAK oligomerization, VDAC channel formation, and stress-induced changes in mitochondrial membrane permeability, and determines the timing and intensity of inflammatory signals. At the disease level, this “enhanced encoding” mtDNA signal has been verified in various cardiometabolic diseases, and its immune effect is closely related to the oxidation state and functional activity of mtDNA (14).Yuzefovych et al. (2019) found that the circulating mtDNA level in patients with obesity combined with type 2 diabetes was significantly elevated, and it was positively correlated with the insulin resistance index (HOMA-IR) and chronic inflammatory indicators, suggesting that changes in mtDNA levels are closely coupled with the metabolic inflammatory state. These findings are particularly relevant to metabolic type HFpEF, as in this type, obesity, insulin resistance, and oxidative stress may promote mtDNA release and amplify systemic inflammation through the cGAS-STING-dependent pathway (15). However, there is still limited direct evidence regarding the causal role of mtDNA outflow in initiating or independently promoting the progression of HFpEF. Therefore, this mechanism should be interpreted as a hypothetical mechanism.

### 2.2 mtDNA release under HFpEF-related comorbid stressors

**Figure 2:**
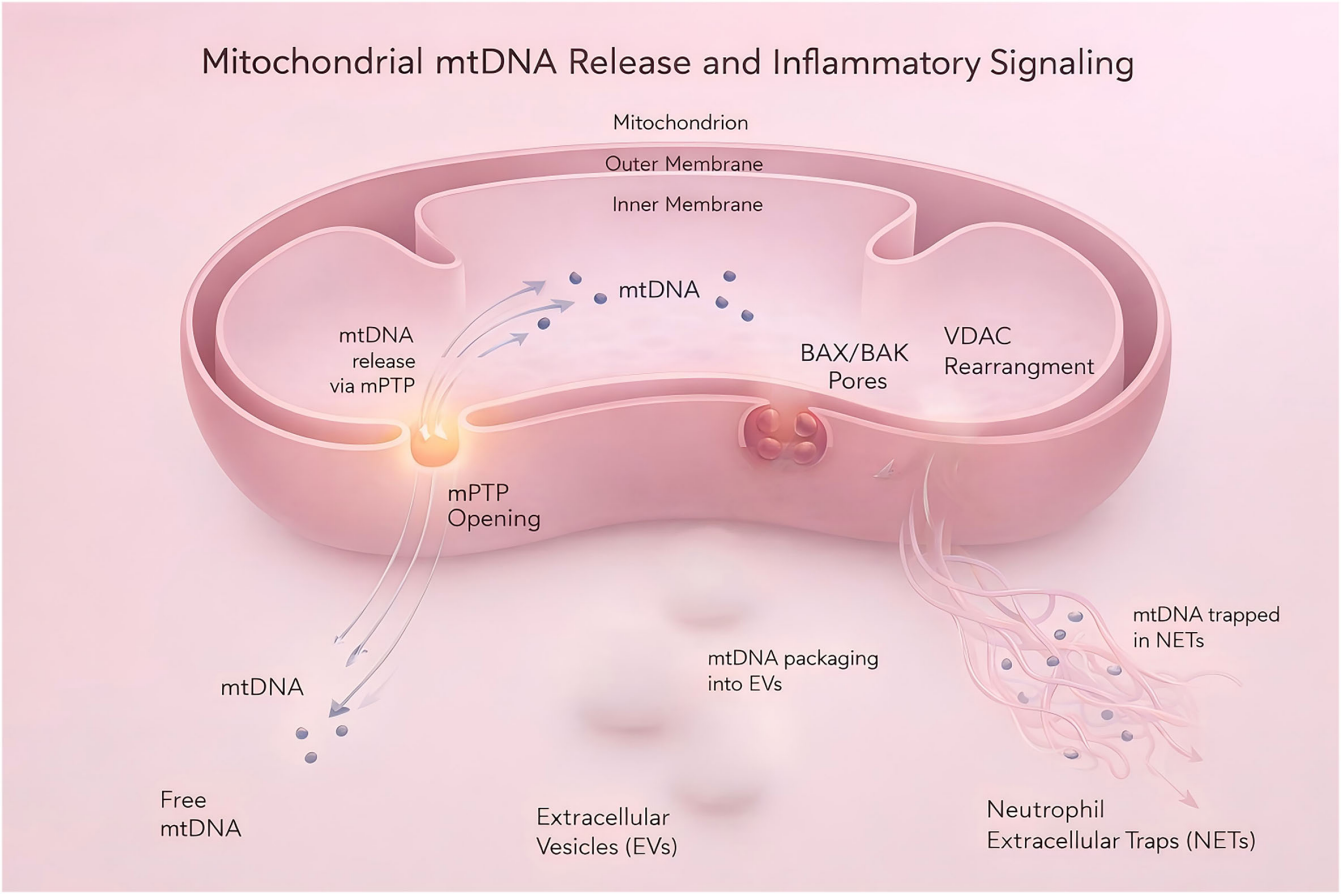
Hierarchical regulatory mechanism of mtDNA ejection. This figure illustrates the key mechanisms by which mitochondrial DNA (mtDNA) is released from the mitochondria and enters the extracellular environment, along with its various transport forms. The center of the figure depicts the mitochondrial structure, including the outer membrane and inner membrane, with mtDNA located in the mitochondrial matrix. Under stress conditions, the mitochondrial permeability transition pore (mPTP) opens (the illuminated area on the left), allowing mtDNA to move from the matrix into the intermembrane space and further migrate outward through increased inner membrane permeability. Subsequently, the outer membrane permeability is further enhanced through the formation of pores by the oligomerization of BAX/BAK and the rearrangement of voltage-dependent anion channels (VDAC) structure, thereby facilitating the entry of mtDNA into the cytoplasm. The released mtDNA can enter the extracellular environment through various pathways (below): some exist in a free form; some are packaged into EVs; in addition, in an inflammatory state, the neutrophil extracellular trap can capture and carry it. The arrows in the figure represent the migration paths of mtDNA from the mitochondria to the cytoplasm and the extracellular space, and different branches represent their different release and transport modes. mtDNA, mitochondrial DNA; mPTP, mitochondrial permeability transition pore; BAX, Bcl-2-associated X protein; BAK, Bcl-2 antagonist/killer; VDAC, voltage-dependent anion channel; EVs; NETs, neutrophil extracellular traps.

The release of mtDNA from the mitochondria into the cytoplasm and extracellular space is a coordinated active regulatory process that is jointly mediated by BAX/BAK pores, VDAC oligomerization, and mPTP membrane permeability, rather than a passive leakage. The hierarchical regulatory mechanism for mtDNA extrusion is shown in **Figure 2**. This process can be summarized into three consecutive stages: endoplasmic release, extracellular membrane permeability, and cytoplasmic accumulation. The changes in the permeability of different membrane structures, driven by stress signals, form a coordinated system with a temporal sequence and functional division of labor (16). The mechanism of mtDNA release and the transmembrane pathways are shown in **Table 1**. At the initial stage, the mitochondrial membrane permeability transition (mPTP) responds to oxidative stress or calcium overload, allowing mtDNA to move from the matrix to the intermembrane space (17); subsequently, the oligomerization of BAX/BAK forms large pores and rearranges with the VDAC channel, promoting mtDNA crossing the outer membrane and entering the cytoplasm (16).McArthur et al. (3) observed in primary fibroblasts using live-cell lattice light-sheet microscopy and cryo-electron microscopy that after BAX/BAK activation, macropores with diameters of approximately 30–700 nm were formed, which allowed the mitochondrial inner membrane protrusion and the release of intact mtDNA (3); Kim et al. (14) further discovered that inhibiting VDAC oligomerization could reduce mtDNA release by approximately 50 - 60%, while inhibiting mPTP only decreased it by 30 - 40% (14), suggesting that the outer membrane channels have a more direct role in the release efficiency. These pathways form a dynamic coupling system: the opening of mPTP can promote VDAC aggregation and stabilize the BAX/BAK pore structure, thereby maintaining the continuous release of mtDNA, explaining the phenomenon of low-level continuous efflux under chronic metabolic stress (18).

**Table 1:** mtDNA release mechanisms and transmembrane pathways.

Release mechanism	Key protein/channel	Trigger condition	Contribution to cytosolic/circulating mtDNA	Temporal feature	Feedback loop	Notes/quantitative info	Reference
Inner Membrane Permeabilization (mPTP)	CypD	Oxidative stress, Ca²^+^ overload	Promote mtDNA into intermembrane space	Transient/Short-term	Upstream signals enhance outer membrane permeability	~30–40% increase in mtDNA release	(17)
Outer Membrane Pore Formation	BAX/BAK Oligomerization	ROS, metabolic stress	Promote mtDNA into cytosol	Low-level continuous	Amplifies efflux with VDAC cooperation	Macropores 30–700 nm	(3)
VDAC Oligomerization	VDAC Oligomers	Mechanical stress, obesity	Increase mtDNA efflux efficiency	Chronic	Maintains BAX/BAK pore stability	Inhibition reduces mtDNA release by 50–60%	(14)
Cytosolic Accumulation & Clearance	PINK1–Parkin Mitophagy	Aging, obesity, HFpEF	Balance cytosolic mtDNA levels	Chronic	Mitophagy defect → mtDNA accumulation → activates STING	Mitophagy defect increases mtDNA 2–3-fold	(19)

mtDNA, mitochondrial DNA; mPTP, mitochondrial permeability transition pore; CypD, cyclophilin D; BAX/BAK, Bcl-2 family pore-forming proteins; VDAC, voltage-dependent anion channel; PINK1, PTEN-induced kinase 1. “Contribution” refers to the role of each mechanism in mtDNA translocation from mitochondria to cytosol or circulation. Quantitative values represent approximate ranges reported in representative studies and may vary across experimental models. Temporal features indicate relative dynamics (transient versus chronic) rather than absolute duration. Mechanistic pathways are not mutually exclusive and may act cooperatively under pathological conditions.

**Figure 3:**
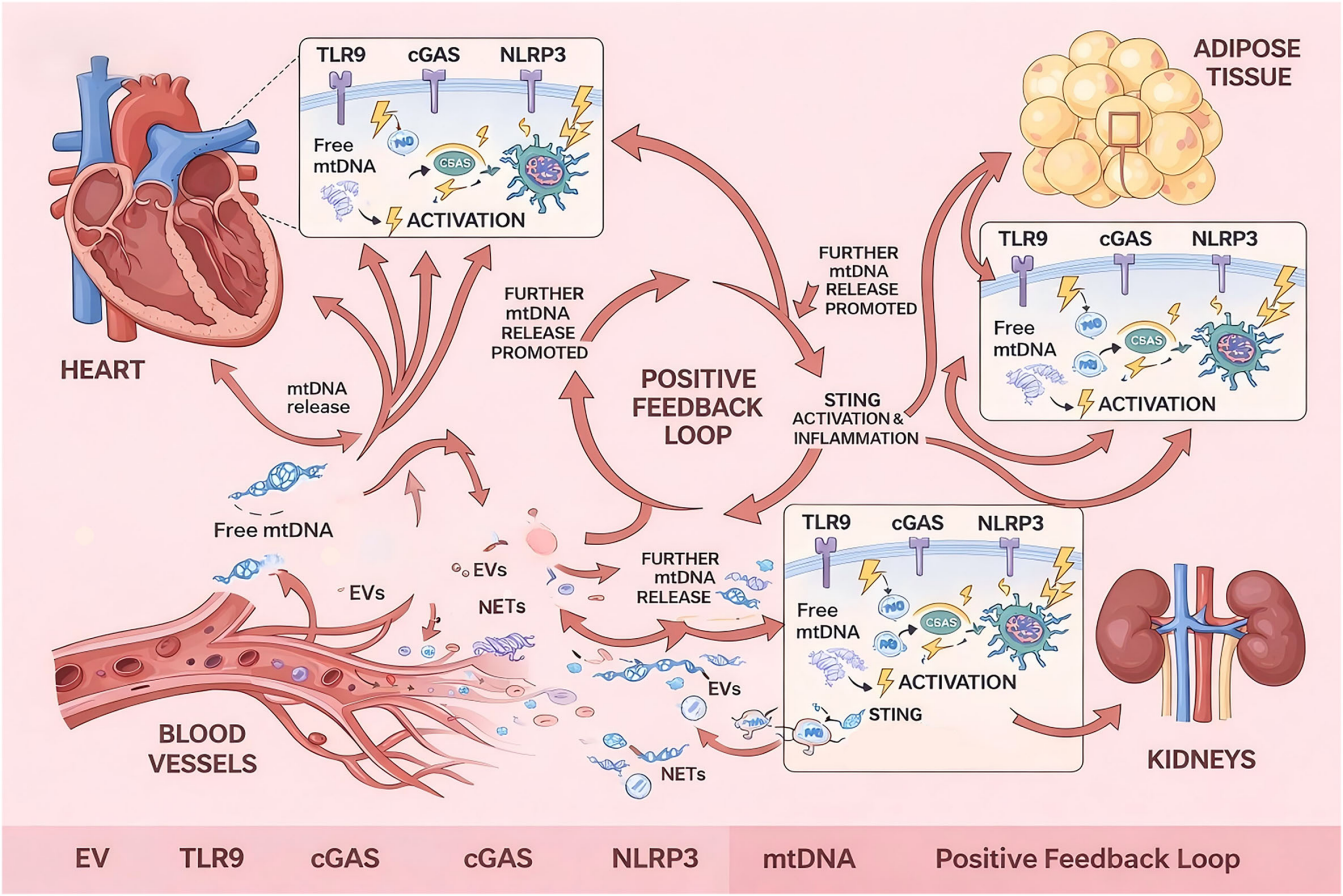
Mechanism diagram of mtDNA trans-cellular propagation and systemic diffusion. This figure illustrates a proposed mechanism by which mtDNA may spread between multiple organs and participate in a positive feedback inflammatory network. Under mitochondrial damage or stress conditions, mtDNA is released from tissues such as the heart into the circulation system (left, “mtDNA release”). In the blood vessels, mtDNA exists in various forms, including free mtDNA, mtDNA encapsulated in EVs, and mtDNA carried by NETs (lower vascular region). The mtDNA in the circulation is taken up or recognized by cells in different organ tissues (heart, adipose tissue, and kidney modules).Within each tissue cell, mtDNA can be recognized by nucleic acid sensors such as Toll-like receptor 9 (TLR9) and cyclic GMP-AMP synthase (cGAS), while NLRP3 acts as the downstream inflammasome platform to amplify the inflammatory output (activation), accompanied by enhanced inflammatory responses mediated by the STING pathway. These signals jointly promote local inflammatory responses and changes in cellular functions. The activation of inflammation further promotes the continuous release of mtDNA (“further mtDNA release promoted”), forming a central “positive feedback loop” centered on mtDNA. The arrows in the figure represent the propagation paths of mtDNA between different organs and the amplification process of inflammatory signals, with bidirectional and circular arrows emphasizing the network characteristics of cross-organ information exchange and self-enhancement. mtDNA, mitochondrial DNA; EVs; NETs, neutrophil extracellular traps; TLR9, Toll-like receptor 9; cGAS, cyclic GMP-AMP synthase; STING, stimulator of interferon genes; NLRP3, NOD-like receptor family pyrin domain containing 3.

After mtDNA enters the cytoplasm, its fate depends on the balance between “removal” and “recognition”. PINK1-Parkin-mediated mitochondrial autophagy can limit its accumulation, while in states related to HFpEF such as aging and obesity, autophagy impairment causes mtDNA to persist and be perceived by the immune system (19). STING activation can also reverse mitochondrial autophagy, forming a positive feedback loop of “release - accumulation - re-release” (20).Although many of the mechanistic data come from non-HFpEF models, such as myocardial ischemia-reperfusion injury or chronic kidney disease, these data are relevant to HFpEF because similar mitochondrial stress, impaired mitochondrial autophagy, and chronic oxidative damage also exist in HFpEF-related comorbid conditions (16). Based on this, some mtDNA can further enter the extracellular environment, expanding intracellular signals into transmissible inflammatory information. The biological activity of circular mtDNA is not solely determined by its abundance; it is also influenced by its extracellular packaging form and transport mode, such as free mtDNA, protein-complex-bound mtDNA, and extracellular vesicle (EV)-encapsulated mtDNA. The transmembrane propagation of mtDNA and systemic diffusion are shown in **Figure 3**.

In HFpEF, circulating mtDNA may mainly exist in the forms of free DNA, protein-complex-bound DNA, and extracellular vesicle (EV)-encapsulated DNA. Each of these forms may differ in terms of stability, tissue distribution, and inflammatory potential. These forms exhibit significant differences in stability, distribution, and cellular uptake mechanisms (21). The trans-organ transmission of mtDNA and the forms of the carriers are shown in **Table 2**.The half-life of free mtDNA is relatively short, but it can be rapidly recognized by TLR9 and induce acute inflammation (22); in contrast, mtDNA encapsulated in EVs is more stable and can enter the distal cells through receptor-mediated endocytosis, thereby preferentially activating the cGAS-STING pathway and maintaining a chronic inflammatory state (23). Studies have also found that EVs from different sources have certain tissue targeting properties. For example, EVs derived from adipose tissue are more easily taken up by endothelial cells, while EVs from immune cells tend to act on the bone marrow and spleen (24). NETs also serve as important carriers, capable of carrying oxidized mtDNA and playing a key role in the spread of inflammation (25).This cell-specific response is the core of the heterogeneity of HFpEF, as endothelial cells, cardiomyocytes, fibroblasts, macrophages, adipocytes, renal cells and skeletal muscle cells may decode the same mtDNA signal into different pathological outputs.

**Table 2:** mtDNA inter-organ transport and extracellular packaging form.

Extracellular packaging and mode of transport	Stability	Organ tropism	Signal preference	Immunogenic activity	Function	Temporal feature	Reference
Cell-free DNA	Short	Broad	TLR9	High	Induce transient inflammation	Transient	(22)
Extracellular Vesicle (EV)	High	Endothelial/Immune Cells	cGAS–STING	Medium-High	Maintain chronic inflammation, inter-organ transport	Chronic	(5)
Neutrophil Extracellular Traps (NETs)	Moderate	Inflammatory sites	TLR9/cGAS	Medium	Carry oxidized mtDNA, amplify local signal	Intermediate	(4)
Protein Complex	Moderate	Broad	cGAS–STING	Low	Extend signal duration, support systemic integration	Chronic	(25)

mtDNA, mitochondrial DNA; EVs, extracellular vesicles; NETs, neutrophil extracellular traps; TLR9, Toll-like receptor 9; cGAS, cyclic GMP-AMP synthase; STING, stimulator of interferon genes. Carrier properties (stability, organ tropism, and signal preference) represent predominant trends observed in experimental systems. Immunogenic activity and functional annotations are context-dependent and may vary with disease state, cellular origin, and mode of mtDNA packaging. Temporal features indicate relative persistence of signaling (transient, intermediate, or chronic) rather than absolute duration. Different carrier forms are not mutually exclusive and may act coordinately in systemic inflammatory propagation.

## 3 HFpEF-relevant mtDNA sensing and inflammatory amplification

In the mtDNA-related innate immune signaling pathway, it is crucial to distinguish between the nucleic acid sensors and the inflammasome platform. TLR9 and cGAS are true nucleic acid sensors, capable of recognizing DNA rich in CpG in the endolysosomal compartment and double-stranded DNA in the cytoplasm, respectively. In contrast, NLRP3 should not be regarded as a direct mtDNA receptor. Instead, NLRP3 mainly functions as a scaffold for inflammasome formation and a downstream amplification platform for inflammation. Mitochondrial stress, reactive oxygen species (ROS) generation, potassium ion efflux, lysosomal damage, and oxidized or ectopically localized mtDNA may all promote the activation of the NLRP3 inflammasome, leading to caspase-1 activation, the maturation of IL-1β/IL-18, and pyroinflammatory amplification. Therefore, in this review, TLR9 and cGAS-STING are described as the mtDNA-responsive nucleic acid sensing pathway, while NLRP3 is described as the inflammasome platform and the downstream amplifier of mtDNA-related inflammatory signals.

### 3.1 Compartmentalized nucleic acid recognition: mechanisms and roles

In HFpEF, mtDNA-related inflammation is regulated both spatially and temporally: TLR9 in the endosome and cGAS-STING in the cytoplasm act as nucleic acid sensing systems, capable of initiating or integrating mtDNA-related signals; while the NLRP3 inflammasome serves as a downstream amplification platform, enhancing caspase-1 activation, IL-1β/IL-18 maturation, and compartment-specific inflammatory responses. The first step is the recognition at the endosome level. When mtDNA enters the endosome, TLR9 is usually the earliest sensing system to be mobilized (26). Due to the rich un-methylated CpG sequences in mtDNA and its greater susceptibility to oxidation modification, it can rapidly activate signaling pathways such as NF-κB, thereby inducing the expression of inflammatory factors like IL-6 and TNF (27). In the state of elevated circulating mtDNA, the TLR9-related signals often rise synchronously with the levels of inflammatory factors and are consistent with changes such as endothelial activation and immune cell recruitment (28). That is to say, TLR9 is more like a “quick starter”: it reacts quickly, but it relies on continuous mtDNA input; otherwise, the signal is difficult to maintain. More importantly, TLR9 can also upregulate the expression of NLRP3 and pro-IL-1β, providing “pre-activation” conditions for the subsequent activation of the inflammasome (29). Therefore, TLR9 is not only responsible for the initial recognition here, but actually determines whether the entire system enters a state that can be further amplified.

**Figure 4:**
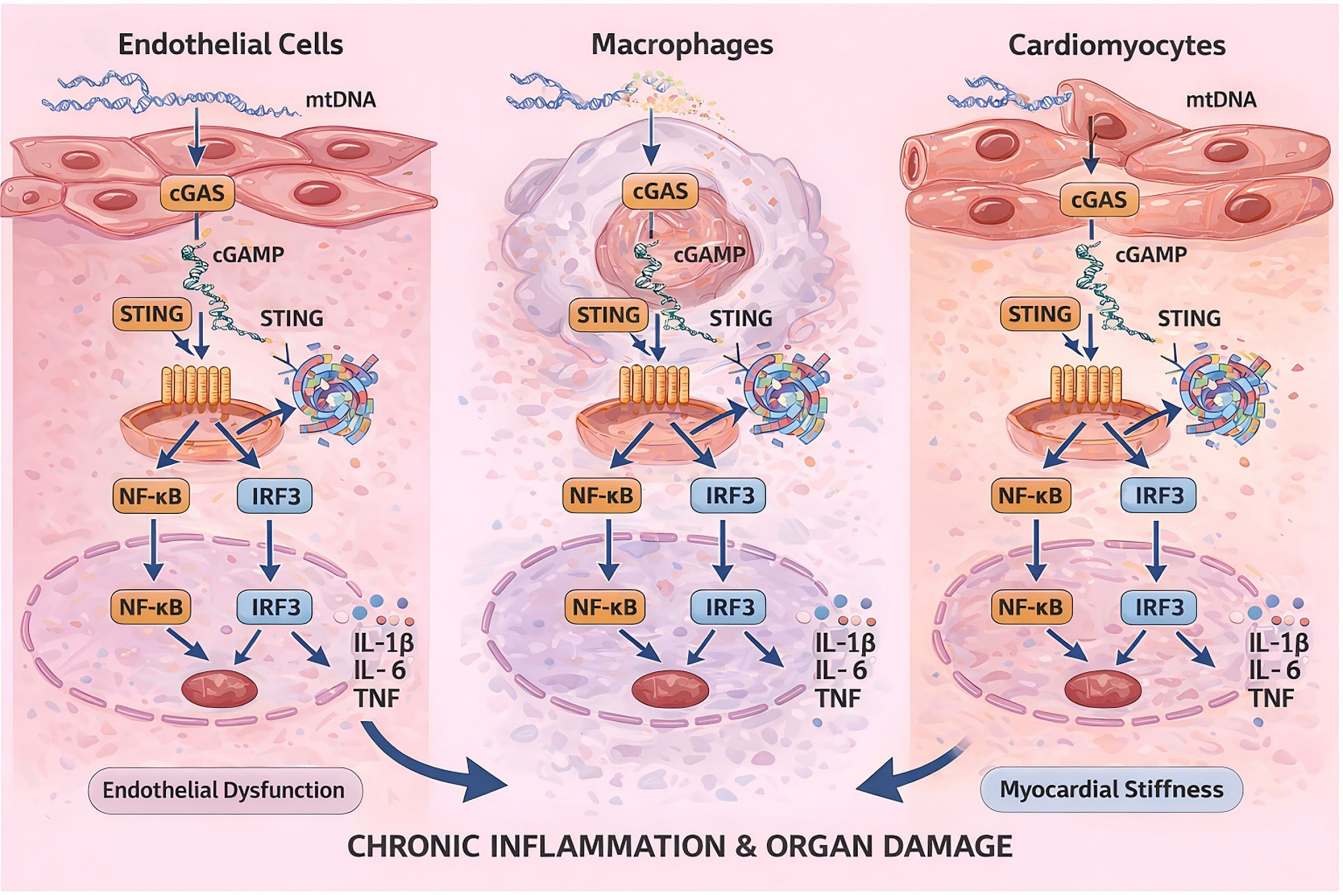
Hierarchical perception of mtDNA and mechanism of inflammatory signal amplification. This figure illustrates the mechanism by which mtDNA induces inflammatory responses and organ damage in different cell types through the cGAS–STING signaling pathway. From left to right in the figure are endothelial cells, macrophages, and cardiomyocytes. In each type of cell, mtDNA entering the cytoplasm is first recognized by the cyclic GMP-AMP synthase (cGAS) and catalyzes the production of the second messenger cGAMP. cGAMP further activates the STING protein on the endoplasmic reticulum, inducing conformational changes and downstream signal transduction. Activated STING recruits and activates related kinases, respectively, to initiate the NF-κB and IRF3 signaling pathways (in the middle). NF-κB and IRF3 then translocate to the nucleus, promoting the transcription of inflammatory-related genes. In the nucleus, these signaling pathways induce the expression of various inflammatory factors, including IL-1β, IL-6, and TNF (at the bottom). These inflammatory mediators produce different pathological effects in different cell types: endothelial dysfunction in endothelial cells; amplification of inflammatory responses in macrophages; and promotion of myocardial stiffness and structural remodeling in cardiomyocytes. The arrows in the figure represent the direction of signal transduction, and the parallel structure of the three cell types emphasizes the common mechanism and cell-specific effects of this pathway in different tissues. The bottom arrow indicates that these processes may jointly contribute to chronic inflammation and organ damage (chronic inflammation & organ damage). mtDNA, mitochondrial DNA; cGAS, cyclic GMP-AMP synthase; cGAMP, cyclic GMP-AMP; STING, stimulator of interferon genes; NF-κB, nuclear factor kappa B; IRF3, interferon regulatory factor 3; IL, interleukin; TNF, tumor necrosis factor.

When mtDNA further enters the cytoplasm, cGAS–STING can function as an intrinsically context-dependent signaling module in mtDNA-related inflammatory signals. This step usually relies on endosomal rupture, mitochondrial-derived vesicles, or EV-mediated delivery (30). After mtDNA enters the cytoplasm and binds to cGAS, it generates cGAMP, which then activates STING and continuously induces the expression of interferons and a series of inflammatory genes through TBK1 and IRF3 (31). Zuo et al. (32) found in a chronic epilepsy model caused by neuron-specific Mic19 knockout that chronic mitochondrial damage can lead to continuous mtDNA leakage, and this process is positively correlated with the continuous activation of the cGAS-STING pathway in microglia, rather than short-term fluctuations; after applying the STING inhibitor H-151, the related inflammatory phenotypes were significantly alleviated (32). Although this study was not conducted in an HFpEF model, it provides mechanistic support for a concept directly related to HFpEF: cGAS-STING may be particularly sensitive to the low but persistent mtDNA input generated by chronic comorbid stress. Compared to TLR9, STING is more like a “slow variable integrator”: it does not necessarily require a strong instantaneous stimulus, but can accumulate small signals continuously into a stable inflammatory output (33). In addition, STING can further upregulate the expression of NLRP3 and related molecules through NF-κB, increasing the cell’s responsiveness to subsequent stimuli (34).Therefore, in the context of chronic low-grade inflammation in HFpEF, cGAS–STING may function as an integrative node that is context-dependent, helping to maintain specific inflammatory outputs and connecting mtDNA-related inflammatory modules. However, this does not mean that STING is the universal primary regulatory factor for inflammation in HFpEF, as parallel existing pathways such as TLR9, NLRP3 inflammasomes, metabolic stress, cellular senescence, complement, and cytokine-related pathways may provide redundancy or compensatory inflammatory inputs. The mechanism of mtDNA perception and signal amplification is shown in **Table 3**. Under the background of continuous stress and signal accumulation, the NLRP3 inflammasome takes on the role of final amplification and execution. Unlike TLR9 and cGAS-STING, NLRP3 is more dependent on the “pre-activation” in the early stage and changes in the intracellular environment, rather than directly recognizing mtDNA as a single ligand (35). Oxidative stress related to mtDNA, calcium ion imbalance and mitochondrial dysfunction will jointly promote the assembly of the inflammasome, thereby activating caspase-1 and releasing effect molecules such as IL-1β and IL-18 (36). In various inflammatory and cardiovascular disease models, the accumulation level of mtDNA, the activation level of NLRP3, and the degree of tissue damage often change synchronously (37). More importantly, once the inflammasome is activated, it will further increase mtDNA release through processes such as cell damage, changes in membrane permeability, and even pyroptosis, thus forming a positive feedback loop (38).The induction of inflammation by mtDNA is not a single step but a dynamic process that progresses step by step: TLR9 initiates quickly, cGAS–STING integrates and maintains it continuously, and NLRP3 is responsible for the final amplification and execution of the effect (39). The hierarchical perception of mtDNA and the mechanism of inflammation signal amplification are shown in **Figure 4**. This hierarchical perception structure may explain how chronic mitochondrial damage in the organs associated with HFpEF transforms into persistent endothelial inflammation, myocardial remodeling, renal-cardiac interaction, and peripheral tissue dysfunction.

**Table 3:** mtDNA-responsive nucleic acid sensing and NLRP3 inflammasome amplification mechanisms.

Signal node	Functional module	Activation trigger	Downstream pathway	Local inflammatory output	Systemic metabolic effect	Temporal feature	Feedback loop	Primary target organ	Reference
TLR9	Endosome	Unmethylated CpG mtDNA	NF-κB → IL-6/TNF	Transient cytokine release ↑2–4 fold	Endothelial adhesion molecules ↑	Transient	Upregulates NLRP3/pro-IL-1β ↑	Endothelial, immune cells	(27)
cGAS–STING	Cytosol	mtDNA fragments/EV delivery	TBK1–IRF3, NF-κB → IFN/inflammatory cytokines	Sustained low-grade inflammation	ROS↑, ATP↓20–40%, Ca²^+^ homeostasis perturbation	Chronic	Promotes mtDNA release & NLRP3 upregulation ↑	Heart, endothelium, immune cells	(11)
NLRP3 inflammasome	Inflammasome platform/downstream inflammatory amplifier	Mitochondrial stress, ROS, K^+^ efflux, Ca²^+^ dysregulation, lysosomal injury, oxidized or mislocalized mtDNA-associated signals	Caspase-1 → IL-1β/IL-18	Inflammation amplification, fibrosis induction	Exacerbate mitochondrial dysfunction, metabolic disturbance	Chronic	Further promotes mtDNA efflux ↑	Heart, kidney, immune tissues	(35)

mtDNA Sensing and Signal Amplification Mechanism. mtDNA, mitochondrial DNA; ROS, reactive oxygen species; IFN, interferon; IL, interleukin; TNF, tumor necrosis factor; EVs, extracellular vesicles; TLR9, Toll-like receptor 9; cGAS, cyclic GMP-AMP synthase; STING, stimulator of interferon genes; NLRP3, NOD-like receptor family pyrin domain containing 3. Pathway annotations represent predominant signaling cascades but may involve additional intermediates depending on cellular context. Quantitative changes (e.g., fold increase or percentage variation) are approximate ranges derived from representative studies. Temporal features indicate relative signaling dynamics (transient versus chronic). Feedback loops denote regulatory trends and may differ across disease states and organ systems. Signaling pathways are interconnected and may act sequentially or cooperatively in inflammatory amplification.TLR9 and cGAS are nucleic acid receptors that can respond to mtDNA, while NLRP3 is not a direct mtDNA receptor. Here, NLRP3 is listed as a platform for the formation of inflammasomes and a downstream amplifier of inflammation; in the context of mitochondrial stress, ROS generation, ion imbalance, lysosomal damage, and mtDNA-related signals with oxidative or mislocalized localization, NLRP3 can be activated. Although cGAS-STING is classified as an integration module, this does not mean that STING is a universal “master regulator” of inflammation in HFpEF. Its functional importance is expected to be context-dependent and may vary depending on mtDNA load, interferon activity, degree of mitochondrial damage, disease stage, and the specific subtype of HFpEF.

### 3.2 Context-dependent STING-mediated integration: mechanisms and functional roles

In HFpEF, the significance of STING is not merely “identifying mtDNA”, but lies in its role in determining whether inflammatory signals can transform from transient stimuli to persistent pathological states. Therefore, STING is discussed here as a HFpEF-relevant inflammatory integrator rather than as a general DNA-sensing molecule. When mtDNA enters the cytoplasm, cGAS binds to it and generates cGAMP, which subsequently activates STING (40). Activated STING, on the one hand, induces interferon expression through the TBK1-IRF3 axis, and on the other hand, promotes the production of various inflammatory factors through NF-κB (41). In the cardiovascular and metabolic conditions associated with HFpEF, STING-related signals typically co-occur with elevated levels of circulating mtDNA, IL-6, TNF, and chemokines (37). These changes are often accompanied by the recruitment of immune cells, endothelial dysfunction, and the expansion of tissue inflammation, suggesting that STING has the ability to translate local mtDNA signals into systemic inflammatory output. Unlike TLR9, which is more inclined to initiate rapidly, STING is more sensitive to “low-level but persistent” mtDNA input, and thus STING signals can be maintained for a long time even with a low stimulation intensity (42). This is highly consistent with the chronic low-grade inflammation characteristics of HFpEF.

The more crucial aspect of STING is that it not only receives signals but also reversely reinforces the upstream damage, thereby connecting inflammation with mitochondrial dysfunction into a self-sustaining process (43).Mun et al (44). In THP-1 macrophages induced by PMA differentiation, combined with rotenone treatment, mitochondrial ROS detection, membrane potential and ultrastructural analysis, cytoplasmic mtDNA quantification, and STING inhibitor H-151 and mPTP inhibitor cyclosporine A intervention, found that STING activation could significantly increase mitochondrial ROS and promote mPTP opening, thereby promoting the release of oxidized mtDNA and the activation of inflammasomes (44). This means that STING is not merely located downstream of mtDNA but participates in the formation of a positive feedback loop of “mtDNA release - STING activation - further mitochondrial damage - more mtDNA release”. At the same time, STING can also upregulate NLRP3 and pro-IL-1β through NF-κB pathways, making cells more prone to enter the inflammasome activation state (44). In many models, the activation degree of STING is consistent with the expression of NLRP3 and IL-1β release (45) indicating that it does not operate independently but plays a connecting and amplifying role between different inflammatory modules. More specifically, STING can also change the response threshold of cells, making them more sensitive to subsequent stimuli, so even with a weak subsequent input, it can trigger a significant inflammatory output (46). This is precisely why in intervention studies, inhibiting STING can reduce inflammatory factor levels, reduce immune cell infiltration, and improve tissue function, but rarely allows the system to fully recover, suggesting that there are redundancies and bypass compensations within this network.

Viewed in the overall context of HFpEF, this “continuous integration + positive feedback” effect of STING has greater systematic significance. Comorbid conditions such as obesity, diabetes, and aging will long-term increase mitochondrial stress, leading to the continuous release of mtDNA; and once this low-level input persists, STING can remain activated in various cell types (47). In endothelial cells, it promotes the expression of adhesion molecules and chemokines, enhancing the recruitment of immune cells (48); in immune cells, it maintains the pro-inflammatory phenotype and promotes the continuous release of inflammatory factors (49). In cardiac muscle cells, it is more associated with metabolic disorders, cellular stress and structural remodeling (50).Therefore, in HFpEF, STING is more appropriately understood as a system amplifier and signal integration module that depends on specific circumstances, rather than a simple nucleic acid sensing receptor, nor a “master regulatory factor” applicable to all situations. It can integrate weak and persistent cytoplasmic DNA-related signals into a continuous inflammatory output, especially in the presence of mitochondrial damage, cytoplasmic mtDNA accumulation, and interferon-rich innate immune activation. However, this effect should be understood in the context of the extensive redundancy of the HFpEF inflammatory network. Even if the STING activity is weakened, the coexisting TLR9-dependent endosomal DNA sensing, NLRP3 inflammasome activation, metabolic stress signals, senescence-associated inflammation, complement activation, neurohormonal pathways, and cytokine networks may still maintain the inflammatory state. Therefore, the observation that a single downstream cytokine blockade is usually insufficient in efficacy does not imply that STING is a single-node solution for all HFpEF phenotypes. On the contrary, a strategy targeting STING may be most applicable to the HFpEF phenotypes with higher mtDNA levels or those dominated by mitochondrial damage, and it may need to be combined with other intervention measures, such as reducing mtDNA release, improving mitochondrial quality control, limiting the amplification effect of inflammasomes, or addressing metabolic and kidney-related comorbidities.

### 3.3 Inflammatory amplification: coupling with metabolic reprogramming

In HFpEF, mtDNA-related inflammation and metabolic imbalance jointly act on key disease phenotypes, including impaired endothelial nitric oxide signaling, insufficient energy supply in cardiomyocytes, abnormal calcium handling, fibrosis, and decreased exercise tolerance. In the context of obesity, diabetes, and aging, mitochondrial dysfunction, increased reactive oxygen species, and energy metabolism disorders often occur simultaneously with elevated circulating mtDNA levels, and are positively correlated with IL-6, TNF, and various chemokines (51). This phenomenon suggests that mtDNA is not only an “readout indicator” of mitochondrial damage, but also participates in maintaining the inflammatory state itself. At the mechanistic level, mtDNA can induce inflammatory responses through TLR9 and the cGAS-STING-mediated nucleic acid sensing, and can further promote the amplification of inflammation dependent on the NLRP3 inflammasome; and these inflammatory signals, in turn, will aggravate mitochondrial dysfunction. This inflammatory-metabolic coupling is particularly prominent in immune cells, and its essence is that the metabolic state directly participates in regulating the “amplification efficiency” of inflammatory signals. Gomes’ research indicates that in infection models, STING activation can increase succinate accumulation and mitochondrial ROS, stabilize HIF-1α and drive macrophages to a glycolytic phenotype (52); while Ma et al. (50) further discovered in the metabolic heart failure model that the increase in mitochondrial ROS induced by lipotoxicity and the continuous release of mtDNA are closely related to the continuous activation of the cGAS-STING pathway and the production of inflammatory factors such as IL-1β, IL-18, and TNF (50). These results collectively point to a key point: immune cells do not respond passively to mtDNA signals, but rather convert them into continuous inflammatory output through metabolic reprogramming. At the same time, free fatty acids released by adipose tissue and the local hypoxic environment further stabilize this high-response state, making it more difficult for the system to return to the baseline (53). Therefore, at this level, immune cells actually act as an “amplifier”, continuously translating metabolic abnormalities into inflammatory signals and, in turn, exacerbating mitochondrial damage.

In HFpEF, the inflammatory signals induced by mtDNA are coupled with metabolic stress, which may lead to impaired calcium handling, reduced ATP production, lack of endothelial nitric oxide (NO), and diastolic dysfunction, rather than just elevated cytokine levels. Persistent mtDNA and inflammatory signals make cardiomyocytes more prone to energy supply deficiency, abnormal calcium handling, and diastolic dysfunction (54), which is highly consistent with the core phenotype of HFpEF; at the same time, endothelial cells show weakened nitric oxide signaling, increased adhesion molecules and chemokines, and impaired microcirculation function. These changes usually occur simultaneously with elevated circulating mtDNA, increased inflammation, and tissue remodeling, rather than existing independently. Therefore, in HFpEF, a more reasonable understanding is that the amplification of inflammation and metabolic imbalance are two sides of the same process, with The former provides a continuous supply of inflammation, while the latter provides the cellular environment necessary to sustain this input. It is this bidirectional coupling that makes inflammation exhibit a low but persistent characteristic and promotes the coordinated remodeling of the heart, blood vessels, and peripheral tissues.

## 4 mtDNA-related inflammatory amplification in cardiac remodeling: mechanisms and functional consequences

### 4.1 Endothelial dysfunction and microcirculatory remodeling: mechanisms and consequences

In HFpEF, endothelial dysfunction is one of the earliest and most critical pathological changes. In specific disease contexts, mtDNA-related inflammatory signals may promote this process by amplifying endothelial inflammation and oxidative stress. Clinical studies have provided important evidence for this association. Fetterman et al. (55) found that in patients with atherosclerosis and/or diabetes, the degree of mtDNA damage in peripheral blood mononuclear cells(PBMCs) was approximately 2–3 times higher than that in healthy controls, and it was significantly correlated with increased vascular pulsatility and decreased endothelial-dependent dilation function (55). Although this study was not limited to HFpEF, it supports the existence of a connection between mtDNA damage and vascular dysfunction that is related to HFpEF, especially because endothelial dysfunction and microvascular inflammation are the core features of HFpEF. From the molecular mechanism perspective, mtDNA that enters the cytoplasm or circulation can be taken up by endothelial cells and recognized by cGAS to generate cGAMP, thereby activating STING (56). STING then induces the expression of inflammatory genes through the dual pathways of TBK1-IRF3 and NF-κB, and directly regulates endothelial metabolism and dilation function: on the one hand, it inhibits phosphorylation at the eNOS Ser1177 site and promotes uncoupling, reducing the bioavailability of NO by approximately 30-50%; on the other hand, it enhances mitochondrial ROS generation and inhibits the Nrf2 antioxidant pathway, further amplifying the oxidative stress level (57).The proposed “mtDNA–cGAS–STING–NO axis” may promote inflammatory activation at the molecular level and reconfigure the redox state of the endothelium and the vasodilatory capacity of blood vessels. transforming endothelial cells from metabolic steady-state regulators to inflammatory signal response units.

Recent studies have further suggested that in HFpEF, this process may involve persistent inflammation activation maintained by continuous mitochondrial stress and mtDNA-related signals, and it is closely related to specific disease contexts. In HFpEF, mtDNA-related STING activation is not a transient inflammatory event but is more likely to constitute a continuously amplified axis for maintaining chronic endothelial damage and microcirculation dysfunction (58). Shi et al. (59) found in the HFpEF model that mtDNA release related to cellular senescence can maintain chronic inflammation in the endothelium through the STAT1-STING axis and promote vascular stiffness, and SGLT2 inhibitors can significantly improve microvascular function by inhibiting this axis, suggesting that STAT1 may be a key amplification node that converts transient DNA sensing into a continuous inflammatory program (59). Yun et al. (60) further suggested that mtDNA-related STING activation may contribute to cardiovascular aging and microcirculatory dysfunction in HFpEF, indicating that STING not only mediates inflammatory signals but may also promote endothelial transformation from a steady-state regulator to an inflammatory effector cell by exacerbating oxidative stress, inhibiting eNOS function, and reducing NO bioavailability (60). In metabolic-related HFpEF, this process further presents a cross-organ propagation feature: EVs derived from adipose tissue carrying mtDNA can be preferentially taken up by endothelial cells and continuously activate the STING pathway (61–64), thereby establishing an “metabolic tissue → vessel” information input path and explaining the widespread systemic microvascular dysfunction in HFpEF. Therefore, from a systemic perspective, the oxidized-modified mtDNA fragments (approximately 500–650 bp) can be released through the BAX/BAK–VDAC channel and serve as pathological signals for organ-to-organ transmission; the imbalance between their continuous release and clearance can be amplified through the STING-mediated pathway, causing endothelial damage and forming a self-sustaining cycle: mtDNA input – STING activation – ROS generation – endothelial dysfunction – further mtDNA release.

### 4.2 Myocardial stiffness and diastolic dysfunction: mechanisms and pathophysiological consequences

Myocardial stiffness and diastolic dysfunction are the core phenotypes of HFpEF. Their formation is not solely driven by structural remodeling, but stems from the long-term imbalance of coupling between inflammatory signals and metabolic stress. During this process, the continuous mtDNA-related signals may help maintain the stress state within cardiomyocytes through the cGAS–STING pathway (65). Zhao et al. (65) found that the level of circulating free mtDNA is significantly positively correlated with the activation of the cGAS-STING pathway (including cGAS, TBK1, IRF3 and their phosphorylation levels) as well as the expression of inflammatory factors (65). This suggests the crucial role of this pathway in chronic inflammation integration. From the molecular mechanism perspective, mtDNA activating STING not only induces inflammatory responses through NF-κB, but also inhibits the activity of complex I/III of the electron transport chain through TBK1-mediated mitochondrial protein phosphorylation, reducing ATP production by approximately 20 - 40% and accompanied by a decrease in mitochondrial membrane potential and an increase in ROS generation (66–68);The latter further forms a positive feedback loop of “ROS- mtDNA damage-STING reactivation”. Additionally, the latest research indicates that STING can enhance ER-mitochondria intercellular Ca²^+^ transport by regulating mitochondrial-associated endoplasmic reticulum membranes(MAMs), thereby integrating metabolism and signal responses at the subcellular level (69). In HFpEF, this suggests that the mtDNA-STING axis may link inflammation, energy metabolism and cellular stress responses; however, the extent to which this pathway directly causes diastolic dysfunction associated with human HFpEF remains to be clarified.

At the functional level, the continuous release of mtDNA may lead to a metabolic-calcium homeostasis imbalance by promoting ATP deficiency (reducing SERCA2a-mediated calcium recycling), ROS-mediated RyR2 dysfunction, and titin modification, collectively producing metabolic–calcium homeostasis imbalance. On one hand, the reduction in ATP production limits the calcium recycling process dependent on SERCA2a, causing the delay in cytosolic Ca²^+^ clearance during diastole (70); on the other hand, ROS-mediated oxidative stress can alter the open probability of RyR2 and reduce its stability, while modifying myofilament proteins(such as titin), reducing myofilament compliance, thereby increasing passive stiffness and prolonging diastolic time (71). Moreover, inflammatory signals can also change the expression levels of calcium regulatory proteins through NF-κB and STAT pathways, further solidifying this process (72). In HFpEF, these changes are not triggered by acute injury but accumulate gradually in a long-term low-level inflammatory environment: continuous but low-level mtDNA release maintains chronic activation of STING, keeping cardiomyocytes in a metastable stress state of “low ATP-high ROS-calcium handling disorder”, rather than leading to cell death (73–75). Therefore, in HFpEF, mtDNA may not only affect myocardial function through inflammatory signals, but also exert its effects by influencing metabolism and calcium homeostasis, thereby contributing to the development of a stable phenotype characterized by myocardial cell stiffness and impaired diastolic function, rather than transient functional disorders.

### 4.3 Fibrosis and immune microenvironment reprogramming: mechanisms and systemic effects

In HFpEF, the mtDNA-amplified inflammatory response is more like a self-sustaining system network rather than a single-point abnormality of a certain pathway. Both clinical and experimental studies have suggested that elevated circulating mtDNA is often synchronized with the activation of inflammatory pathways (76). Bae et al. found that the plasma circulating free mtDNA level was significantly increased in patients with type 2 diabetes and was consistent with the increase in IL-1β; further *in vitro* experiments showed that patient-derived mtDNA could directly activate the AIM2 inflammasome in macrophages and promote the mature secretion of IL-1β and IL-18 (77), suggesting that mtDNA may function not only as a DAMP but also as a contributor to inflammatory amplification (78). From the perspective of molecular mechanisms, continuous mtDNA input may activate a hierarchical innate immune response, which involves TLR9 and cGAS-STING-mediated nucleic acid sensing, as well as the amplification effect of the downstream NLRP3 inflammasome: TLR9 initiates rapid inflammation through endosomes, while cGAS-STING integrates continuous cytoplasmic DNA signals and upregulates the expression of pro-IL-1β through the TBK1-IRF3 and NF-κB axes (79–82), And ROS, ion imbalance and oxidative mtDNA synergistically promote the assembly of the NLRP3 inflammasome, resulting in an approximately 2-4-fold increase in the secretion of mature IL-1β (10). Moreover, the STING signal can enhance TGF-β expression through IRF3 and non-classical pathways and promote fibroblast activation and collagen deposition through paracrine mechanisms, thereby establishing a direct molecular connection between inflammation and fibrosis (83–86).In the past three years, further studies have further demonstrated that this process is closely related to immune metabolic reprogramming: mtDNA-related signaling can promote a shift of macrophages toward a glycolysis-dependent pro-inflammatory phenotype (glycolysis ↑ by approximately 30-60%), and inhibit oxidative phosphorylation, thereby maintaining the persistence of inflammatory output (87).

More importantly, this system has significant self-reinforcing and structural fixation characteristics. The inflammatory response further promotes mtDNA release through increased ROS, aggravated mitochondrial damage, and changes in membrane permeability(such as mPTP opening and VDAC rearrangement), causing the cytoplasmic mtDNA level to continuously increase and re-activate STING and inflammasomes, thus forming a positive feedback loop of “mtDNA input - signal integration - inflammation amplification - re-release” (12, 43). In HFpEF-related models, STING activation not only enhances inflammation but can also induce myocardial hypertrophy and fibrosis through the macrophage-fibroblast axis (59). In cardiorenal syndrome, the mtDNA clearance ability is decreased(such as DNase activity reduced by approximately 30-50%), which can lead to its continuous accumulation in the circulation and promote the amplification of inflammatory and fibrotic signals through STING-NLRP3 in a synergistic manner (88–90). This mechanism is associated with HFpEF, as chronic kidney disease is a common comorbidity of HFpEF, which may increase the circulating mtDNA load and thereby enhance the renal-cardiac inflammatory crosstalk. Therefore, from the systemic perspective, fibrosis and immune microenvironment remodeling are not the terminal results of inflammation but the “structural fixation” of mtDNA information flow at the tissue level, This indicates a transition of HFpEF from an inflammatory amplification state to a more stable pathological network state, which is also the key reason why the disease is difficult to reverse.

## 5 mtDNA-associated cross-organ inflammatory networks and HFpEF heterogeneity: mechanisms and implications

In HFpEF and its associated comorbidities, the increase in mtDNA is often not an isolated event but occurs concurrently with enhanced inflammation, metabolic abnormalities, and changes in organ function, presenting a directionally consistent dynamic change (91). In obesity-related HFpEF, fat tissue inflammation, mitochondrial stress, and elevated circulating mtDNA are often accompanied by endothelial dysfunction and microcirculation disorders (15, 92, 93); in diabetes-related HFpEF, enhanced oxidative stress, disrupted fatty acid metabolism, and increased mtDNA release are coupled with deterioration of myocardial diastolic function (37, 94–96); and in the combined setting of chronic kidney disease and heart failure with preserved ejection fraction (HFpEF), impaired mtDNA clearance may increase the circulating mtDNA load and exacerbate the renal-cardiac inflammatory crosstalk, thereby promoting persistent inflammation and structural remodeling (97). Oka et al. (6) discovered through the construction of a mouse model with myocardial cell-specific deficiency of lysosomal deoxyribonuclease II(DNase II) that mtDNA, due to autophagy degradation, accumulates abnormally in the cytoplasm and mediates inflammatory responses through TLR9, inducing fibrosis and heart failure (6). This suggests that mtDNA not only changes concurrently with inflammation and organ damage, but also may play a causal role in inflammation under certain experimental conditions. However, current evidence is mainly based on cross-sectional associations and lacks longitudinal analysis of the temporal sequence. During the progression of HFpEF, whether mtDNA functions as an initiating signal or mainly acts as an amplifier of existing inflammation remains to be clarified. This distinction is crucial because multiple non-mtDNA mechanisms can independently participate in the pathogenesis of HFpEF. The impairment of the endothelial NO–cGMP–PKG pathway, coronary microvascular inflammation, low phosphorylation of myosin, extracellular matrix expansion, arterial stiffness, cardiomyocyte hypertrophy, abnormal sodium handling in the kidneys, neurohormonal activation, decreased metabolic flexibility, and reprogramming of immune cells may all contribute to the HFpEF phenotype without the need for mtDNA escape as the primary initiating event.

Therefore, the mtDNA-related signals should be regarded as an interaction level within the broader HFpEF network. Depending on the background of comorbidities, tissue compartments, and disease stages, it may act upstream, downstream, or concurrently with these established mechanisms.

At the mechanism level, recent studies have gradually developed a more systematic explanatory framework: the biological effects of mtDNA are not solely determined by its presence, but are jointly determined by its source, carrier form, cellular entry pathway, nucleic acid sensing pathway, inflammasome amplification module, and the coupling relationship between downstream effect responses. MtDNA from different tissue sources differ in release mode, oxidation state, and damage characteristics, thereby affecting its immune recognition efficiency; for example, mtDNA from adipose tissue is more likely to participate in metabolic inflammation (98),while mtDNA from the myocardium is more associated with energy imbalance and systolic/diastolic abnormalities (99). At the same time, the form of transportation determines its scope of action and persistence: free mtDNA is prone to degradation, The mtDNA encapsulated within extracellular vesicles (EVs) has higher stability and can be transported across organs, preferentially activating the cytoplasmic cGAS-STING sensing mechanism; moreover, depending on the different cellular stress backgrounds, it can also promote the amplification effect of the downstream NLRP3 inflammasome (37, 100, 101).Furthermore, the differences in the expression of nucleic acid receptors, adaptor molecules, components of the inflammasome, and downstream effector pathways among different cell types will result in different output effects of the same mtDNA signal in endothelial cells, immune cells, and cardiomyocytes. Within this framework, TLR9 and cGAS-STING mainly mediate early recognition and continuous signal integration, while NLRP3 mainly mediates inflammation amplification dependent on the inflammasome (102–104). This “source-carrier-receptor-pathway” chain provides an explanation for how mtDNA signals are selectively amplified in multiple organs, but most of these are indirect inferences from different models and lack direct verification of the signal propagation path in a unified system.

Based on this, mtDNA-related signals may help explain part of the heterogeneity of HFpEF, especially the inflammatory components and cross-organ communication components within it; however, it should be integrated with the established mtDNA-independent mechanisms that shape various phenotypes. Recent studies have shown that elevated circulating mtDNA is consistent with increased inflammatory factors, endothelial dysfunction, and tissue remodeling in various populations (7), but the key difference does not lie in the intensity of inflammation, the key lies in the distinction of the “source - perception - response” framework: In this framework, the source and molecular state of mtDNA determine how it is perceived by the innate immune pathway and further transformed into organ-specific inflammatory and remodeling responses. In metabolic-related HFpEF, mtDNA derived from adipose tissue and associated with extracellular vesicles (EVs) may be transmitted between organs and activate STING-related signals, thereby amplifying immune-metabolic reprogramming and abnormal systemic energy utilization (37).;In age-related HFpEF, low but persistent mtDNA input may preferentially maintain chronic microvascular inflammation through TLR9 and STING-related pathways, although the dominant receptor involved remains to be determined (6).; In cardiorenal syndrome, the limited clearance of mtDNA leads to its continuous accumulation in the circulation and the synergistic amplification of inflammation and fibrosis through STING and NLRP3 (91);In vascular-dominant HFpEF, endothelial dysfunction, arterial stiffness, impaired ventricular-vascular coupling, and the lack of NO–cGMP–PKG may occur through numerous mtDNA-independent mechanisms; nonetheless, in certain circumstances, the mtDNA–STING signal may still exacerbate endothelial inflammation and microcirculation dysfunction (57). Therefore, these phenotypes should be regarded as overlapping network states, shaped by the amplification of mtDNA-related inflammation and the non-mtDNA-dependent disease mechanisms together; the relative contributions of each pathway will vary depending on the individual patient and the stage of the disease. Therefore, the relative contribution of mtDNA-related signals in different phenotypes of HFpEF is unlikely to be uniform. In metabolic HFpEF, obesity, insulin resistance, lipotoxicity, and adipose tissue inflammation may induce mitochondrial stress and mtDNA release, making mtDNA-related innate immune activation as an inflammatory amplifier of particular significance. In aging-related HFpEF, impaired mitochondrial autophagy, cellular senescence, and inflammatory senescence may promote the accumulation of cytoplasmic or circulating mtDNA. In cardiorenal HFpEF, renal dysfunction, congestion, uremic toxins, and decreased nucleic acid clearance capacity may increase the systemic burden of mtDNA-related damage-associated molecular patterns (DAMPs). In contrast, in vascular-dominant or hypertension-related HFpEF, arterial stiffness, pressure load, endothelial nitric oxide (NO) deficiency, ventricular-vascular coupling imbalance, and extracellular matrix remodeling may play a dominant role, while mtDNA-related signals may only exert a secondary or context-dependent amplification effect. When interpreting mtDNA as a biomarker, mechanism mediator, or therapeutic target for HFpEF, such phenotypic-specific differences should be fully considered.

More importantly, this heterogeneity has a dynamic evolutionary feature. As the burden of comorbidities increases, the levels of circulating mtDNA and inflammation continue to rise, and the signal coupling between different organs gradually strengthens (2). Once a pathway becomes dominant in a specific organ, it can further promote mtDNA escape by enhancing oxidative stress, cell damage, or EV release, forming a positive feedback amplification across organs. For example, EV-mtDNA derived from adipose tissue can act on skeletal muscle and cardiomyocytes and induce mitochondrial dysfunction, thereby increasing mtDNA release (105); In the state of impaired renal function, the clearance obstacles cause mtDNA to accumulate continuously and repeatedly stimulate the heart and blood vessels, thereby stabilizing the inflammatory phenotype (57, 91). Therefore, HFpEF can be regarded as a systemic cross-organ inflammatory syndrome, in which mtDNA-related signals may help maintain and amplify the inter-organ crosstalk, and should not be merely considered as a disease confined to the myocardium. Viewing HFpEF as a stratifiable and transformable “network state” helps explain its heterogeneity and provides a framework for mechanism-oriented precise classification and intervention. The key to the future lies in clarifying the causal and temporal sequence roles of mtDNA escape in the transmission of inflammation across organs. and the “input-integration-output” process, and using longitudinal cohorts and spatial omics methods to analyze its dynamic flow. Based on this, multi-target combined intervention strategies should be verified in clinical relevant models to promote the transition from single-pathway treatment to systemic network regulation. The relative positioning of mtDNA-related and non-mtDNA mechanisms across representative HFpEF phenotypes is summarized in **Table 4**.

**Table 4:** Relative positioning of mtDNA-related and non-mtDNA mechanisms across HFpEF phenotypes.

HFpEF phenotype	Key trigger/condition	Major non-mtDNA mechanisms	mtDNA-related mechanism	Contribution to HFpEF heterogeneity	Temporal/functional feature	Reference
Metabolic HFpEF	Obesity, diabetes, insulin resistance, lipotoxicity	Adipose inflammation, metabolic inflexibility, endothelial dysfunction, systemic low-grade inflammation	Mitochondrial stress may promote mtDNA release; EV-associated or circulating mtDNA may engage TLR9- and cGAS–STING-mediated nucleic acid sensing and promote downstream NLRP3 inflammasome amplification	Amplifies immune-metabolic remodeling and systemic inflammation	Chronic amplifier downstream of metabolic stress	(113–115)
Aging-related HFpEF	Aging, cellular senescence, impaired proteostasis	Inflammaging, vascular stiffening, impaired autophagy/mitophagy, mitochondrial quality-control failure	Defective mitophagy and mitochondrial damage may increase cytosolic or circulating mtDNA, thereby activating innate immune pathways	Links mitochondrial aging with sterile inflammation and innate immune activation	Chronic, slowly progressive inflammatory amplifier	(6, 116, 117)
Cardiorenal HFpEF	CKD, congestion, uremic toxins, RAAS activation	Sodium-handling abnormalities, renal inflammation, volume overload, neurohormonal activation, systemic oxidative stress	Renal dysfunction and congestion may increase systemic inflammatory burden and impair clearance of mtDNA-associated DAMPs	Promotes renal–cardiac inflammatory crosstalk, but is unlikely to be the sole amplifier of cardiorenal HFpEF	Context-dependent; stronger with renal dysfunction, congestion, or systemic inflammation	(2, 114, 118)
Vascular-dominant HFpEF	Arterial stiffness, endothelial dysfunction, microvascular rarefaction	NO–cGMP–PKG impairment, coronary microvascular inflammation, ventricular–vascular uncoupling, endothelial dysfunction	mtDNA-responsive TLR9 and cGAS–STING signaling may aggravate endothelial and macrophage activation	May contribute to microvascular inflammation, but non-mtDNA vascular mechanisms may dominate	Secondary or context-dependent amplifier	(114, 118, 119)
Hypertensive HFpEF	Pressure overload, LV hypertrophy, arterial stiffening	Fibroblast activation, ECM deposition, myocardial stiffness, titin hypophosphorylation, concentric remodeling	Oxidative mitochondrial injury under pressure overload may promote mtDNA release and innate immune activation	Reinforces inflammatory fibrosis and myocardial remodeling rather than serving as the primary initiator	Remodeling amplifier rather than primary disease trigger	(6, 118, 119)
Inflammatory/multimorbid HFpEF	Multiple comorbidities, systemic inflammation, immune dysregulation	Immune-cell reprogramming, cytokine networks, metabolic–renal–vascular interaction, comorbidity clustering	Circulating mtDNA may act as a DAMP linking injured tissues and organs through nucleic acid-sensing pathways and downstream inflammasome amplification	Supports cross-organ inflammatory network remodeling and may be particularly relevant in highly inflamed endotypes	Network-level inflammatory amplifier	(2, 114, 120)

TLR9 and cGAS-STING are regarded as nucleic acid sensing pathways that can respond to mtDNA, while NLRP3 is considered a downstream inflammasome amplification platform and is not a direct mtDNA receptor. mtDNA, mitochondrial DNA; HFpEF, heart failure with preserved ejection fraction; CKD, chronic kidney disease; RAAS, renin-angiotensin-aldosterone system; EV, extracellular vesicles; DAMP, damage-associated molecular pattern; ECM, extracellular matrix; NO–cGMP–PKG, nitric oxide–cGMP–protein kinase G. This table summarizes the representative evidence supporting the mtDNA-related mechanisms and non-mtDNA mechanisms in different phenotypes of HFpEF. It does not imply that mtDNA is the dominant mechanism in all HFpEF phenotypes. On the contrary, mtDNA-related signals are positioned as an inflammatory amplifier with phenotypic and stage-dependent properties within a broader HFpEF pathological biology network. The STING-related mechanism should be understood as an integrated module that depends on specific circumstances, rather than a universally applicable therapeutic bottleneck; its relevance may be most prominent in HFpEF subtypes with higher mtDNA levels, increased interferon levels, or being dominated by mitochondrial damage.

## 6 Targeting mtDNA-related inflammatory amplification: mechanisms, therapeutic strategies, and clinical implications

In HFpEF, mtDNA-related inflammatory signals may represent a mechanism through which chronic comorbid stress is transformed into persistent inflammatory input and feedback amplification. Therefore, the treatment strategy needs to shift from a single anti-inflammatory approach to network-level regulation. Recent studies have observed a consistent trend in animal models and human populations: elevated circulating mtDNA is often accompanied by increased inflammatory factors, decreased endothelial function, and aggravated tissue remodeling, and shows similar directional changes in different backgrounds such as obesity, diabetes, and chronic kidney disease (7, 15, 37, 106). Meanwhile, in intervention studies, inhibiting a single inflammatory factor often only temporarily reduces inflammatory levels and is difficult to prevent disease progression, suggesting that there is still a continuous upstream input in the system (98, 107). From a mechanistic perspective, a positive feedback loop is formed between mitochondrial damage, mtDNA escape, STING/NLRP3 activation, and further mitochondrial stress: mtDNA, as a signal, is perceived on the one hand, and on the other hand, it continuously promotes new mtDNA release through enhanced oxidative stress and damage processes (10). This “continuous input + feedback amplification” structure enables inflammation to persist for a long time under low-intensity conditions. Therefore, a potential therapeutic strategy for HFpEF should be to reduce mtDNA release, weaken the activation of mtDNA sensing pathways, and enhance mtDNA clearance, rather than merely targeting a single downstream cytokine. The important point is that this network-level perspective does not imply that any single upstream molecule can completely overcome the redundancy of the inflammatory network. On the contrary, candidate upstream nodes such as STING should be understood as regulatory factors that depend on specific contexts, and their therapeutic value depends on the dominant inflammatory module in a particular subtype of HFpEF.

However, given the heterogeneity of the mechanisms underlying HFpEF, treatments targeting mtDNA are unlikely to have a consistent therapeutic effect for all patients. Their efficacy may depend on whether a particular patient or subtype has increased mitochondrial damage, accumulation of mtDNA in the circulation or tissues, activation of the TLR9 or cGAS-STING nucleic acid sensing pathway, and amplification of the downstream NLRP3 inflammatory body. Therefore, mtDNA-targeted strategies should be regarded as complementary to other treatments rather than alternatives; these other treatments include interventions targeting hemodynamic load, endothelial dysfunction, renal function impairment, metabolic diseases, neurohormonal activation, and extracellular matrix remodeling.

In the treatment framework centered on HFpEF, the goal of upstream intervention is to reduce mtDNA leakage and restore mitochondrial homeostasis in key tissues closely related to the disease, including the myocardium, endothelium, adipose tissue, kidneys, skeletal muscle, and immune cells. Studies have shown that in HFpEF and its related comorbidities, mitochondrial autophagy decline, increased oxidative stress, and changes in membrane permeability often occur simultaneously with increased mtDNA release, and are consistent with the enhanced STING and NLRP3 signals (37, 97). Restoring PINK1-Parkin-related autophagy, reducing ROS levels, and stabilizing mitochondrial membrane structure can reduce cytoplasmic and circulating mtDNA load and accompany a decrease in inflammatory signals (108). In various models, such interventions usually simultaneously reduce STING activity, inflammatory factor expression, and the degree of tissue damage.

At the middle and downstream levels, the intervention focus shifts to regulating signal integration and preventing the “fixation” of inflammation. cGAS-STING may function as an upstream integration module that is context-dependent in mtDNA-related cytoplasmic DNA sensing, participating in the interferon response, NF-κB-related inflammatory gene expression, oxidative stress, autophagy impairment, and secondary mtDNA release (109); However, it should not be regarded as a universal core node for controlling all inflammatory phenotypes of HFpEF. In contrast, NLRP3 mainly determines the inflammatory output dependent on the inflammasome and is closely related to the maturation, fibrosis and structural remodeling of IL-1β/IL-18 (110). Gaidt et al. (111) found through CRISPR/Cas9 gene editing technology in human bone marrow-derived macrophages that knocking out STING or NLRP3 separately and combining with small molecule inhibitors (such as MCC950) in experiments revealed that although inhibiting a single pathway could significantly reduce IL-1β secretion and pyroptosis induced by cytoplasmic DNA, it could not completely block the inflammatory cascade. The study revealed that STING activation induces lysosomal damage, which “compensatorily” triggers NLRP3 inflammasome assembly. This tandem mechanism explains why single-target inhibition often only partially improves the inflammatory state of the tissue, demonstrating the complex redundancy and compensatory effects in the immune regulatory network (111). Although these findings are not specific to HFpEF, they support the rationality of testing the combined strategy of mitochondrial protection and anti-inflammatory in models of HFpEF with metabolic or renal comorbidities. This distinction is helpful in coordinating the redundancy of the inflammatory network with the potential role of STING as a therapeutic node. Redundancy mainly explains why blocking a single downstream cytokine, such as IL-1β, IL-6, or TNF, may not be sufficient in heterogeneous HFpEF populations. STING is different from these downstream mediators because it is located upstream of multiple cytoplasmic DNA sensing outputs, including type I interferon signaling, NF-κB activation, endothelial activation, macrophage polarization, and mitochondrial stress amplification. However, STING should not be regarded as a universally applicable bottleneck node, nor should it be considered a general “master regulatory factor” controlling all inflammatory phenotypes of HFpEF. Its therapeutic relevance may be most prominent in HFpEF subtypes with higher mtDNA levels, interferon enrichment, aging-related factors, or mitochondrial damage as the dominant factor; and when STING is inhibited, the coexisting TLR9 pathway, NLRP3 inflammasome pathway, metabolic pathway, complement pathway, neurohormonal pathway, and aging-related pathway may still continue to maintain inflammation. Therefore, targeting STING-based treatment should be regarded as a subtype-specific strategy that may require combined application, rather than a universal single-drug therapy applicable to all HFpEF phenotypes. Therefore, compared with the sole inhibition of STING, combined and biomarker-guided intervention is more likely to achieve long-lasting efficacy. For instance, it can simultaneously reduce mtDNA load and inhibit STING, improving mitochondrial function while limiting the amplification of NLRP3 inflammasome, or combining mtDNA-targeted therapy with treatments targeting metabolism, kidneys, blood vessels or hemodynamic stress factors. Additionally, the restoration of mtDNA clearance capacity is equally crucial. In cases of impaired renal function and chronic inflammatory states, mtDNA persists in the circulation and shows consistent changes with inflammatory levels and organ damage, suggesting that the clearance impairment itself is involved in the pathological process (91).Enhancing nucleic acid degradation, improving lysosomal function, and restoring the clearance ability of immune cells may shorten the residence time of mtDNA in the body and reduce systemic input (112).

## 7 Future perspectives and conclusion

In the context of HFpEF and its associated comorbidities, recent studies have indicated that the increase in mtDNA is usually accompanied by enhanced inflammation, metabolic dysfunction, impaired endothelial function, and organ remodeling. It also exhibits directionally consistent changes in various backgrounds related to HFpEF, such as obesity, diabetes, and renal insufficiency. This suggests that mtDNA is not specific to HFpEF, but may represent a common mechanism axis. HFpEF-related complications can converge on systemic inflammation and organ dysfunction through this axis. However, most current evidence still mainly comes from cross-sectional association studies, and their temporal position in the disease process is not clear -Whether mtDNA escape plays a role as an early initiation signal or mainly functions as a secondary amplifier to maintain and amplify the existing inflammation in specific situations remains to be further clarified. Therefore, the framework proposed in this review should be regarded as a testable working model rather than established evidence that mtDNA is the main causal amplifying factor in HFpEF. Therefore, the focus of future research should not be on further proving whether mtDNA increases, but on clarifying its causal role in the cross-organ information transmission system. Future research should prioritize the study of phenotypically defined HFpEF cohorts and related models to determine whether the levels of circulating mtDNA, the oxidative state of mtDNA, the extracellular packaging form, and the activation of mtDNA sensing pathways are associated with diastolic dysfunction, congestion, decreased exercise tolerance, renal dysfunction, pulmonary vascular involvement, and clinical outcomes. Single-cell sequencing, spatial transcriptomics, and *in vivo* tracing methods may help clarify whether mtDNA is transferred between the heart, vascular system, adipose tissue, kidneys, skeletal muscle, and the immune system in a causally relevant manner.

Based on this, a key conceptual shift might lie in the following: mtDNA should not merely be regarded as a molecular pattern related to damage or an inflammatory marker, but also as a potential cross-organ inflammatory information carrier and a quantifiable network state variable. The different sources, extracellular packaging forms, and modification states of mtDNA may encode differentiated inflammatory signals, and through the structural coupling of “source - transmission - decoding”, they can generate specific outputs in different cells and organs, thereby promoting the formation of certain inflammatory components and cross-organ components in the heterogeneity of HFpEF. From this perspective, HFpEF is not a disease dominated by a single organ or a single pathway, but rather a systemic pathological state. In this state, the abnormal information flow related to mtDNA may interact with metabolic, vascular, renal, immune, and extracellular matrix mechanisms, jointly promoting the remodeling of the multi-organ network. Therefore, the core of future research lies not only in analyzing individual molecular mechanisms, but also in identifying and regulating the key nodes in this network to break its self-sustaining structure of “continuous input - signal integration - inflammation amplification - re-input”. Future studies should determine whether STING represents a functional bottleneck or merely functions as one of multiple redundant inflammatory modules in specific mtDNA levels that are high or in the interferon-enriched subtype of HFpEF. This distinction is crucial for determining whether STING inhibition should be tested as a standalone strategy or more reasonably as part of a combined treatment guided by biomarkers.

Based on this framework centered on HFpEF, intervention measures targeting mtDNA release, dissemination, perception and clearance may provide new opportunities for mechanism-based classification and individualized treatment of HFpEF, especially for patients with evidence of mitochondrial damage and innate immune activation; however, their clinical efficacy still needs to be directly verified in HFpEF-specific studies.
